# A Novel Chimeric Avidin with Increased Thermal Stability Using DNA Shuffling

**DOI:** 10.1371/journal.pone.0092058

**Published:** 2014-03-14

**Authors:** Barbara Taskinen, Tomi T. Airenne, Janne Jänis, Rolle Rahikainen, Mark S. Johnson, Markku S. Kulomaa, Vesa P. Hytönen

**Affiliations:** 1 BioMediTech, University of Tampere, Tampere, Finland; 2 Fimlab Laboratories, Pirkanmaa Hospital District, Tampere, Finland; 3 Department of Biosciences, Åbo Akademi University, Turku, Finland; 4 Department of Chemistry, University of Eastern Finland, Joensuu, Finland; 5 Tampere University Hospital, Tampere, Finland; University of Alabama at Birmingham, United States of America

## Abstract

Avidins are a family of proteins widely employed in biotechnology. We have previously shown that functional chimeric mutant proteins can be created from avidin and avidin-related protein 2 using a methodology combining random mutagenesis by recombination and selection by a tailored biopanning protocol (phage display). Here, we report the crystal structure of one of the previously selected and characterized chimeric avidin forms, A/A2-1. The structure was solved at 1.8 Å resolution and revealed that the protein fold was not affected by the shuffled sequences. The structure also supports the previously observed physicochemical properties of the mutant. Furthermore, we improved the selection and screening methodology to select for chimeric avidins with slower dissociation rate from biotin than were selected earlier. This resulted in the chimeric mutant A/A2-B, which showed increased thermal stability as compared to A/A2-1 and the parental proteins. The increased stability was especially evident at conditions of extreme pH as characterized using differential scanning calorimetry. In addition, amino acid sequence and structural comparison of the chimeric mutants and the parental proteins led to the rational design of A/A2-B I109K. This mutation further decreased the dissociation rate from biotin and yielded an increase in the thermal stability.

## Introduction

Common to all members of the avidin protein family is their high affinity towards a small ligand, D-biotin. This is the reason why avidin from chicken (AVD) and streptavidin from the bacterium *Streptomyces avidinii* are used in a wide variety of different biotechnology applications [1], [2]. In addition to AVD and streptavidin, several other avidins have also been described including the avidin-related proteins (AVRs) and biotin-binding proteins (BBPs) from chicken [3], [4], bradavidin I and II from *Bradyrhizobium japonicum* [5]–[7], rhizavidin from *Rhizobium etli* [8], shwanavidin from *Shewanella denitrificans* [9], tamavidin from mushroom [10], xenavidin from frog [11], and zebavidin from zebrafish [12]. Despite their relatively low amino acid sequence identity, all avidins share a highly similar tertiary structure, an eight-stranded β-sheet barrel structure, where the biotin-binding site is located at the open end of the barrel. With the exception of rhizavidin, shwanavidin and bradavidin II, all of the structurally characterized natural avidins are stable homotetramers with four biotin-binding sites, one on each monomer. The biotin-binding sites are not equally distributed on each side of the tetramer but instead are organized as pairs on two opposite faces. Even though all avidins have similar tertiary structures, the proteins differ e.g. in their quaternary structure, ligand-binding affinities towards biotin and other small ligands, thermal stabilities and isoelectric points. These differences make the avidin protein family an excellent candidate for the design of novel proteins by DNA family shuffling [13], [14].

We have previously created functional chimeric avidins using DNA shuffled sequences of AVD and avidin related protein 2 (AVR2) [15]. Here, we present the crystal structure of the previously characterized chimeric mutant A/A2-1, which proves that the avidin fold is robust and suitable for extensive genetic manipulation. Furthermore, we have improved the DNA library screening methodology and selected a new mutant, A/A2-B. A/A2-B shares high sequence similarity with A/A2-1, but has a decreased dissociation rate from biotin and a higher thermal stability, exceeding that observed for the highly stable parental protein AVR2. Finally, using rational design, we achieved to further decrease the dissociation rate and increase the thermal stability of A/A2-B by mutating isoleucine 109 to lysine.

## Materials and Methods

### X-ray structure determination of A/A2-1

The vapour diffusion method, 96-well sitting drop iQ plates (TTP Laptech) and TTP Labtech's mosquito liquid handling robot were used to crystallize the A/A2-1 mutant (7.4 mg/ml; 50 mM sodium acetate, pH 4), at 22°C. The protein was mixed with a D-biotin solution (1 mg/ml; 5 mM Tris, pH 8.8, 8 mM CHES, pH 9.5) in a 10∶1 v/v ratio before crystallization. The JCSG-plus Screen (Molecular Dimensions Ltd., Suffolk, UK) gave an initial hit and, after optimization, the A/A2-1 mutant crystals formed typically in 1–2 weeks in drops of protein-biotin solution (300 nl) and well solution (300 nl; 0.09 M phosphate/citrate, pH 4.2; 36% v/v PEG 300); 55 µl of solution was used at the bottom of the well.

X-ray data were collected at the ESRF beam line ID14-1 (Grenoble, France) at 100 K from a single crystal, which was frozen in liquid nitrogen; no cryoprotectant was added. The data were processed with XDS [16] (see Table 1 for statistics) and the phase problem was solved using the molecular replacement program Phaser [17] within the CCP4i GUI [18], [19]. In the molecular replacement, the A chain of AVR2 [PDB: 1WBI] [20] was used as the search ensemble; two chains were searched for. The structure from Phaser was first rebuilt using the automatic procedure of ARP/wARP [21]–[23] and then, in several cycles, manually edited/rebuilt within Coot [24] and refined with Refmac5 [25]. Non-protein atoms were added to the model either with the automatic procedure of Coot and ARP/wARP, or manually in Coot. For structure determination statistics, see Table 1.

**Table 1:** Structure determination statistics for A/A2-1 [PDB: 4BCS].

**Cell parameters**	
Space group	*P*2_1_2_1_2
Unit cell:	
a, b, c (Å)	81.1, 47.2, 60.6
α, β, γ (°)	90, 90, 90
**Data collection** a	
Wavelength (Å)	0.93340
Beamline	ID14-1 (ESRF)
Detector	MarCCD
Resolution (Å)^b^	20-1.8 (1.9-1.8)
Unique observations^b^	22157 (3250)
I/sigma^b^	29.8 (4.7)
*R*_factor_ (%)^b^	4.3 (40.6)
Completeness^b^	99.7 (99.9)
Redundancy^b^	7.2 (7.2)
**Refinement**	
*R*_work_ (%)^c^	18.9
*R*_free_ (%)^c^	22.6
Monomers (asymmetric unit)	2
Protein atoms	1893
Heterogen atoms	53
Solvent atoms	112
*R.m.s.d:*	
Bond lengths (Å)	0.014
Bond angles (°)	1.870

a The numbers in parenthesis refer to the highest resolution bin.

b From XDS [16].

c From Refmac 5 [25].

The final structure of the A/A2-1 mutant was validated using the inbuilt tools of Coot [24], and using MolProbity [26] of the Phenix software suite [27], before depositing the coordinates and structure factors to the Protein Data Bank [28], [29] with PDB entry code 4BCS.

### Selection of A/A2-B by phage display of AVD/AVR2 mutant library

Phage library preparation and biopanning was essentially done as described previously [15]. The chimeric mutants were displayed as a fusion with the pIII coat protein on the surface of M13 bacteriophages. An amber codon between the chimeric gene and the pIII gene allowed simultaneous expression of fusion proteins and free chimeric avidin monomers in amber suppressing *E. coli* strains such as XL1 blue. This resulted in the display of tetrameric avidin proteins on the phage surface [15], [30]. *E. coli* XL1 blue cells harboring the AVD/AVR2 DNA mutant library were infected with VSC-M13 helper phages (Stratagene, La Jolla, CA, USA; 10^11^ pfu/ml) and cultured over night. Phages were collected by precipitation with 20% PEG-6000 in 2.5 M NaCl [15], [31]. The phage titer of purified phages (input titer) was determined by infecting *E. coli* XL1 blue cells with serial dilutions (10^7^ and 10^8^-fold) of the phages for 15 min at 37°C. Phage infected cells were diluted 10-fold and plated on LB plates containing 100 µg/ml ampicillin and 10 µg/ml tetracyclin. Biotinylated BSA and casein used to capture functional biotin-binding proteins were prepared by reaction with EZ-Link Sulfo-NHS-SS-Biotin (Thermo Scientific, Waltham, MA, USA) in 10-fold molar excess in 5 mM HNa_2_PO_4_/H_2_NaPO_4_, 150 mM NaCl, pH 8 for 2 h at room temperature (RT). Unreacted biotinylation reagent was removed by dialysis against PBS. The success of biotinylation was assayed using a HABA assay according to the manufacturer's instructions (Thermo Scientific). Biotinylated proteins in PBS were used to coat MaxiSorp Immuno 96 MicroWell plates (Nunc A/S, Roskilde, Denmark) overnight at 4°C. Biotinylated casein and BSA were used in alternating panning rounds to reduce nonspecific binding to the carrier protein. Precipitated phages were diluted 1∶10 on the first panning round and 1∶2 on the second, third and fourth panning round. Dilutions were made using 1% BSA in PBS in the first and third panning round and 1% casein in PBS in the second and fourth panning round. Diluted phages were incubated over night at 4°C to allow the BSA- or casein-binding phages to bind their respective ligands. Phages (100 µl) were added to wells coated with biotinylated protein (200 ng) and incubated for 90 min with agitation at 600 rpm (Talboys orbital microplate shaker 1000MP, Troemner, Thorofare, NJ, USA) at RT. Wells were washed five times with 0.05% PBS-Tween (PBST) (300 ul). Wells were incubated with PBS (300 µl) containing D-biotin (2 µg) for 15 min at 37°C and 600 rpm in order to elute non-specifically bound phages and phages displaying proteins with a high dissociation rate. Wells were washed with PBS (300 µl) and bound phages were eluted by addition of 50 mM DTT (100 µl) during an incubation of 1 h at 37°C. Eluted phages were used to infect XL1 blue cells and amplified as described in [15]. Phages were subsequently precipitated and the phage titer (output) was determined from 10^2^–10^5^-fold phage dilutions as described above. Master plates were prepared from 22 randomly picked colonies after the fourth panning round. Selected colonies were inoculated into the freezing medium (100 µl of 30 g/L tryptone, 20 g/L yeast extract, 10 g/L MOPS, 0.4 mM MgSO_4_, 36 mM K_2_HPO_4_, 13.2 mM KH_2_PO_4_, 6.8 mM (NH_4_)_2_SO_4_, 1.7 mM sodium citrate, 4.4% glycerol, pH 7) containing 1% (v/v) glucose, 10 mg/ml tetracycline and 50 µg/ml ampicillin. Samples were incubated on 2.2 ml storage plate (ABgene, Thermo Scientific, Surrey, UK) for six hours at 37°C and 600 rpm. In order to increase the total glycerol concentration to 15%, a solution containing freezing medium and glycerol in 1∶1 ratio (24 µl) was added. Plates were shaken for 15 min at 600 rpm and stored at −70°C.

### Avidin-biotin displacement assay

In order to characterize the ligand-binding properties of selected phage colonies, a microplate based protocol was used to get an estimate of the dissociation rate constant for biotin binding. A sample (7 µl) from the master plates prepared after phage panning was used to inoculate SB medium (200 µl) containing 10 µg/ml tetracycline, 100 µg/ml ampicillin and 1% glucose. Cells were grown in a 2.2 ml storage plate (ABgene, Thermo Scientific) for 6 h at 37°C and 600 rpm (Talboys orbital microplate shaker 1000MP, Troemner, Thorofare, NJ, USA) after which the protein expression was induced by adding SB medium (50 µl) containing 5 mM IPTG, 10 µl/ml tetracycine, 50 µg/ml ampicillin and 0.1% glucose. The final concentration of IPTG was 1 mM. Cells were incubated over night at 28°C and 600 rpm. Three MaxiSorp 96 MicroWell plates (Nunc A/S) were coated with BSA (200 ng) in PBS or with a mixture of BSA (50 ng) and BSA-BTN (150 ng) in PBS (200 µl). For each clone to be analysed, one well was coated with BSA and two wells were coated with BSA-BTN. Coating was done over night at 4°C.

Cells were harvested by centrifugation for 10 min at 1,000 g and 4°C. Pellets were frozen at −20°C. Frozen cells were lysed by vigorous shaking in EasyLyse reagent (40 µl) (Epicentre Biotechnologies, Madison, Wisconsin, USA) for 10 min. The cell lysate was diluted with 10 mM Tris-HCl buffer, pH 7.5, 1 M NaCl (200 µl). Cell debris was removed by centrifugation at 8'000 g for 5 min at 4°C. Coated wells were washed 3 times with PBST and blocked with 1% (w/v) milk in PBST (300 µl) for 1 h at RT and then washed again. The clarified cell lysate (70 µl) was added to each well and incubated for 1 h at RT, shaking at 500 rpm (Talboys orbital microplate shaker 1000MP, Troemner). Unbound proteins were washed off with PBST.

In order to detect the bound proteins, biotinylated alkaline phosphatase diluted 1∶5000 in 1% milk in PBST (100 µl) was added to each well. Plates were incubated for 1 h at RT and 500 rpm. After washing, PBS (200 µl) was added to each well of the plate coated with 1% milk in PBS and one plate coated with BSA-BTN. PBS+10 µg/ml biotin (200 µl) was added to each well of the second plate coated with BSA-BTN and incubated for 30 min at RT and 500 rpm. Wells were washed 3 times with PBST and 3 times with PBS. “Phosphatase substrate” (100 µl of 1 mg/ml, Sigma-Aldrich, St. Louis, MO, USA) in DEA buffer (1 M diethanol amine, 0.5 mM MgCl_2_, pH 9.8) was added to each well and the absorbance at 405 nm was measured at 10-minute intervals for one hour using a microplate reader 680XR (Bio-Rad Laboratories Inc., Hercules, CA, USA). The signals were compared between wells treated with free biotin and the untreated wells in order to analyze the rate of biotin displacement instead of just assaying the functionality of the mutants by detection of bound biotinylated alkaline phosphatase. The ratio of the signals gives an estimate for biotin dissociation rate, which is independent of the amount of functional protein expressed.

### QuikChange mutagenesis

QuikChange primers I109K_for (5′-GACTGGAAAGCTACCAGGGTCGGCAACAACGACTTC-3′) and I109K_rev (5′-CCTGGTAGCTTTCCAGTCATCACCAATGTCATTAACACTTG-3′) were designed according to the recommendations of the QuikChange Site-Directed Mutagenesis Kit instruction manual (Statagene) and [32]. A/A2-B phagemid (5 ng) was mixed in a 50 µl reaction with 0.2 mM dNTPs (Thermo Scientific), Pfu buffer+MgSO_4_ (Thermo Scientific),10 pmol of each primer and incubated for 2 min at 90°C. Pfu DNA polymerase (2.5 U, Thermo Scientific) was added, followed by 16 cycles of 30 s at 95°C, 30 s at 55°C and 15 min at 68°C. The PCR product was purified using the GeneJET DNA purification kit (Thermo Scientific) and subsequently digested with 1 µl FastDigest DpnI (Thermo Scientific). The digestion product was purified as described above and transformed into One Shot Top 10 chemically competent *E. coli* cells (Invitrogen). Transformants were plated onto LB plates containing 100 µg/ml ampicillin and the mutated plasmids were isolated from the resulting colonies using GeneJET Plasmid Purification Kit (Thermo Scientific). The expression plasmid was confirmed by sequencing.

### Protein production and purification

The mutants A/A2-1, A/A2-B and A/A2-B I109K in phagemid vector were produced in an *E. coli* bottle culture using BL21star cells and the parental proteins AVD and AVR2 in pET101/D and pBVboostFG, respectively, were produced in BL21-AI cells. BL21star and BL21-AI cells do not suppress the amber stop codon and expression of mutant protein without the pIII fusion is guaranteed. The cells were cultured in 500 ml LB medium containing 100 µg/ml ampicillin at 28°C and 150 rpm to an OD_600_ of 0.25 and induced with 1 mM IPTG and, in the case of BL21-AI, additionally with 0.2% (w/v) L(+)-arabinose. The cells were harvested by centrifugation after overnight expression. Mutant A/A2-1 in phagemid, as well as the parental proteins AVD in pET101/D and AVR2 in pBVboostFG, were additionally produced in *E. coli* BL21-AI using a Labfors Infors 3 fermentor (Infors HT, Bottmingen, Switzerland) as described in [15], [33]. The cells were grown in fermenting medium (4.5 l) containing 100 µg/ml ampicillin and the antifoam agent struktol J647 (Schill+Seilacher, Hamburg, Germany) at 25°C and pO_2_ of 20%. Protein production was induced at an OD_600_ of 20 with 1 mM IPTG alone, or in the case of AVD and AVR2, with 1 mM IPTG and 0.2% (w/v) L(+)-arabinose, and stopped after 24 h. Protein purification by 2-iminobiotin affinity chromatography was performed as described previously [15]. The purified proteins were analyzed with denaturing mass spectrometry to verify their purity and sequence (data not shown).

### Size exclusion chromatography with light scattering analysis

Proteins were analysed using a liquid chromatography instrument (CBM-20A, Shimadzu Corporation, Kyoto, Japan) equipped with autosampler (SIL-20A), UV-VIS (SPD-20A) and fluorescence detector (RF-20Axs) as well as Zetasizer µV light scattering detector (Malvern Instruments Ltd, Worcestershire, UK) for molecular weight (static light scattering) and hydrodynamic size (dynamic light scattering) determination. The instrument was controlled using Lab Solutions Version 5.51 (Shimadzu Corporation) and OmniSEC 4.7 (Malvern Instruments Ltd.). Samples (70 µg in 40–100 µl) were injected on a Superdex75 5/150GL column (GE healthcare, Uppsala, Sweden) equilibrated with Na_2_HPO_4_/NaH_2_PO_4_, 650 mM NaCl, pH 7. Runs were executed with a flow rate of 0.25 ml/min at 12°C. Molecular weight determination was either done by calculating a standard curve of molecular weight markers (cytochrome C, 12.4 kDa; carbonic anhydrase, 29 kDa; ovalbumin, 44 kDa; BSA, 66 kDa, Sigma-Aldrich) or was based on the light-scattering intensity of the eluting protein, for which BSA was used for instrument calibration using the OmniSEC software (Malvern Instruments Ltd.).

### Mass spectrometry analysis

All mass spectrometric (MS) analyses were performed on a 12-T APEX-Qe Fourier transform ion cyclotron resonance (FT-ICR) instrument (Bruker Daltonics, Billerica, MA, USA), interfaced to an electrospray ionisation (ESI) source. The protein sample's buffer was exchanged into 10 mM ammonium acetate (pH 6.9) with the use of PD-10 columns (GE Healthcare). The resulting fractions, which eluted in a volume of 3–4 ml, were pooled and concentrated to approximately 250 µl using Millipore Ultrafree-0.5 Biomax-5 (5-kDa cut-off) centrifugal filter devices (Millipore, Billerica, MA, USA). The concentrations of the protein stock solutions were determined by measuring the UV-absorbance at 280 nm and by using a sequence-derived extinction coefficient calculated using General Protein/Mass Analysis for Windows (GPMAW, Lighthouse Data, Odense, Denmark). In order to analyze the protein under denaturing solution conditions, the stock solution was further diluted with an acetonitrile/water/acetic acid (49.5∶49.5∶1.0, v/v/v, pH 3.2) solvent. Alternatively, the sample was diluted with 10–500 mM ammonium acetate (pH∼7) to perform native-MS analysis. For biotin-binding experiments, D-biotin (Sigma-Aldrich) stock solution (2 mM in water) was mixed at a desired molar ratio with the protein and incubated at RT for 30 min prior to the analysis. For each spectrum, a total of 128–512 co-added 1MWord (128kWord for native-MS) time-domain transients were zero-filled once prior to a fast Fourier transformation, magnitude calculation and external mass calibration with respect to the ions of an ES Tuning Mix (Agilent Technologies, Santa Clara, CA, USA). The instrument was operated and the data were processed with the use of XMASS 6.0.2 software.

### Isothermal titration calorimetry

Estimated biotin affinity to proteins was determined using a high-sensitivity VP-ITC titration calorimetry instrument (Microcal Inc., Northampton, MA, USA) by titrating 75 µM biotin in 15 µl aliquots to 5 µM protein. Measurements were made at 40°C at pH 3 (50 mM sodium citrate, 100 mM NaCl, pH 3) and pH 7 (50 mM NaHPO_4_/Na_2_HPO_4_, 100 mM NaCl, pH 7). Origin 7.0 (Originlab Corporation, Northampton, MA, USA) was used to analyse the data and produce the graphs.

### Dissociation rate determination by fluorescence spectrometry

The dissociation rate constant (k_diss_) of fluorescently labelled biotin was determined by fluorescence spectrometry using the biotin-labelled fluorescent probe ArcDia BF560 (ArcDia, Turku, Finland) as described in [34]. In principle, the changes in the fluorescence intensity of 50 nM dye in 50 mM sodium phosphate, 650 mM NaCl, 0.1 mg/ml BSA, pH 7 was measured after the addition of biotin-binding protein to a final monomer concentration of 100 nM. The dissociation of this complex was observed by addition of a 100-fold molar excess of free biotin. The assay was performed at 25°C using a QuantaMaster Spectrofluorometer (Photon Technology International, Inc., Lawrenceville, NJ, USA) equipped with circulating water bath thermostat. The fluorescence probe was excited at 560 nm and emission was measured at 578 nm.

### Differential scanning calorimetry

The thermal stability of the studied proteins in the presence and absence of ligands was analysed using an automated VP-Capillary DSC System (Microcal Inc.). Thermograms were recorded between 20 and 140°C with a heating rate of 2.0°C/min. Proteins were dialysed either into pH 7 buffer (50 mM NaH_2_PO_4_/Na_2_HPO_4_, 100 mM NaCl, pH 7), pH 3 buffer (50 mM sodium citrate, 100 mM NaCl, pH 3), or pH 11 buffer (50 mM sodium carbonate, 100 mM NaCl, pH 11). Samples were degassed prior to the measurement. The protein monomer concentration in the cell was 30 µM, and the ligand concentration was 105 µM. Results were analysed using the Origin 7.0 DSC software suite (Microcal Inc.).

### SDS PAGE stability assay

The SDS stability assay is based on previously reported assays [35], [36]. To 55 µM protein in pH 7 buffer either a 10-fold molar excess of biotin-5-fluorescein or the same volume of pH 7 buffer was added. Due to the high isoelectric point of avidin, A/A2-1, A/A2-B and A/A2-B I109K (pI>9), the proteins (including AVR2, pI = 4) were acetylated by adding a 40-fold molar excess of Sulfo-NHS-acetate (Thermo Scientific) [35]. The protein sample was divided into equal aliquots and incubated for 20 min at 20, 40, 50, 60, 70, 80, 90, or 100°C. After incubation, the same volume of 2× sample buffer (60 µM Tris-HCl, pH 6.8, 25% glycerol, 0.01% Bromphenol blue) was added, before loading 5 µg of the protein in a 15% acrylamide-bisacrylamide gel. To detect the bound biotin-5-fluorescein, the gels were analyzed under UV in a Molecular Imager Gel Doc XR+ System (BioRad Laboratories, Hercules, CA, USA), before Coomassie Brilliant Blue staining. As a control, proteins were incubated in denaturizing SDS sample buffer (60 µM Tris-HCl, pH 6.8, 25% glycerol, 2% SDS, 0.01% Bromphenol blue, 0.5% β-mercaptoethanol) for 20 min at 100°C. PageRuler Plus Prestained Ladder (Thermo Scientific) was used as molecular weight marker.

### Dynamic light scattering

The hydrodynamic radius of proteins was determined by batch dynamic light scattering using Zetasizer Nano ZS (Malvern Instruments Ltd.). Proteins were dialysed into 50 mM NaH_2_PO_4_/Na_2_HPO_4_, 100 mM NaCl, pH 7) and analysed at a concentration of 1 mg/ml. Three measurements were made with 10 runs of 10 s per measurement. Data was analysed using Zetasizer software v7.01 (Malvern Instruments Ltd). Analysis was based on the volume distribution.

### Miscellaneous methods

The sequence alignment was created using ClustalW [37] and edited using GeneDoc [38] and Microsoft Office Word 2010 (Microsoft). The structural superimpositions were made using the “align” command of PyMOL (The PyMOL Molecular Graphics System, Version 1.5.0.2, Schrödinger, LLC) and the chain A of the A/A2-1 mutant as the reference structure. PyMOL and the visualization and modelling package Bodil [39] was used for visual analyses of the structures. PyMOL was used to create all the figures relating to structural representations. GIMP 2.6.9 and CorelDRAW X5 were used to edit the figures. Theoretical hydrodynamic radius from the obtained crystal structure of the A/A2-1 mutant was estimated using HYDROPRO Version 10 [40].

## Results and Discussion

### Crystal structure of A/A2-1

**Figure 1:**
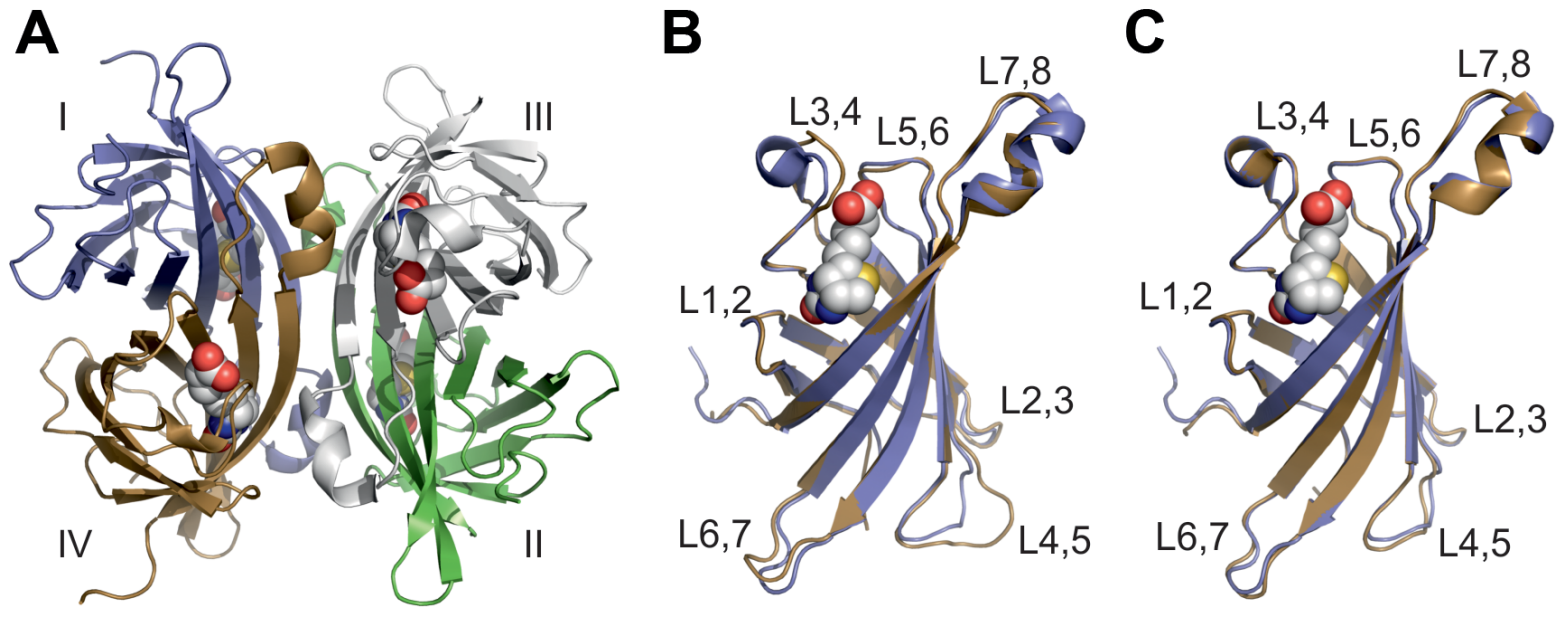
X-ray structure of A/A2-1. A. Cartoon representation of the homotetramer. Subunits I–IV are numbered according to Livnah et al. (1993) [42]: I, blue; II, green; III, light grey; IV, brown. B. Cartoon models of the superimposed A/A2-1 (blue) and chicken AVD (brown; [PDB:1AVD]) subunits I. C. Cartoon models of the superimposed A/A2-1 (blue) and AVR2 (brown; [PDB:1WBI]) subunits I. A–C. The bound biotin ligands are drawn as spheres; carbon atoms are shown in white, sulfur atoms in yellow, nitrogen atoms in blue and oxygen atoms in red. L1,2, the loop between β-strand 1 and 2; L2,3, the loop between β-strand 2 and 3, etc.

**Figure 2:**
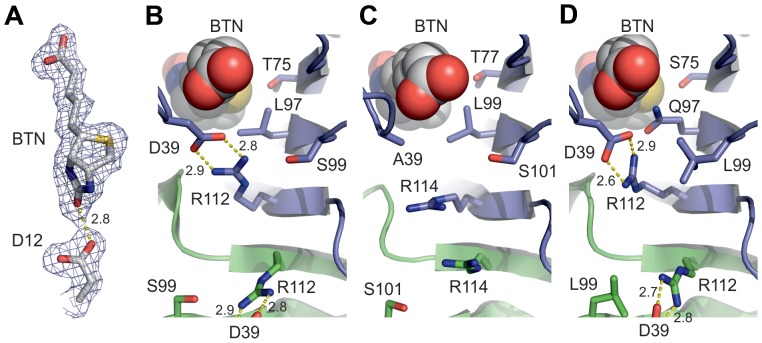
Unique features of A/A2-1. A. A weighted 2F_O_-F_C_ contour map (sigma level 1) showing electron density around biotin (BTN) and D12. The putative hydrogen bond is indicated by a dashed line. B. Salt-bridge between D39 and R112 in the A/A2-1 structure. The side chains of T75, L97 and S99 in the close vicinity of the salt bridge are also shown as stick models; oxygen atoms are shown in red and nitrogen atoms in blue. C. The salt bridge cannot form in the chicken AVD structure [PDB:1AVD] between residues A39 and R114 equivalent to residues D39 and R112 of A/A2-1. Residues T77, L99 and S101 equivalent to T75, L97 and S99 of A/A2-1 are shown. D. Salt-bridge between D39 and R112 in the AVR2 structure [PDB:1WBI]. Residues S75, Q97 and L99 equivalent to T75, L97 and S99 of A/A2-1 are shown. B–D. Cartoon models: subunit I, blue; subunit II green. Biotin (BTN) molecules are shown as spheres; coloring as in Figure 1.

**Figure 3:**
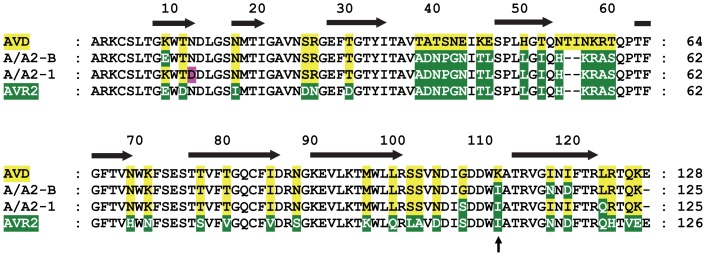
Sequence alignment of AVD, A/A2-1, A/A2-B and AVR2. A/A2-1 and A/A2-B show higher similarity to AVD [NP_990651.1] than to AVR2 [NP_001025519.1]. The two mutants differ from each other only at six amino acid positions. Amino acids originating from AVD and AVR2 are respectively indicated with yellow or green background. A point mutation in A/A2-1 is indicated with pink background. The secondary structure is based on AVD [PDB: 2AVI]; the β-strands are indicated by black arrows. Residue numbering is according to AVD. Isoleucine/lysine residue (111 in avidin, 109 in AVR2, A/A2-1 and A/A2-B) is indicated by a black arrow.

The previously characterized chimeric mutant A/A2-1 showed less tight binding towards biotin as compared to AVD, and displayed higher thermal stability (T_m_ = 92.4°C) in the absence of biotin, but only a small increase in thermal stability was observed upon binding of biotin. In addition, size exclusion chromatography indicated an oligomeric state smaller than a tetramer [15]. Therefore, in order to better understand the structural origin of the physicochemical properties, the structure of mutant A/A2-1 was solved in complex with biotin at 1.8 Å resolution (see Table 1 for the structure determination statistics). Two monomers were found in the asymmetric unit and, as expected based on the high sequence identity to its parental proteins AVD [41] and AVR2 [20], the quaternary structure was homotetrameric (Figure 1A), each monomer having an eight-stranded β-sheet barrel structure with the biotin-binding site located at the open end of the barrel (Figure 1B,C, Figure 2; [42]). When superimposed, A/A2-1 fits better to the AVR2 [PDB: 1WBI] than the AVD structure [PDB: 1AVD], the r.m.s.d values for the displacement of Cα atoms being 0.2 Å and 0.5 Å, respectively. The sequence and structural differences to AVD are in the region derived from AVR2, starting at A38 of the L3,4-loop of A/A2-1 and ending with S58 of the L4,5-loop, whereas A/A2-1 differs from AVR2 at the N- and C-terminal regions originating from AVD (Figure 3).

The individual or cumulative functional effect of the many mutations found in A/A2-1 is not trivial to predict but probably the two most interesting residues of A/A2-1 in terms of ligand binding and stability are D12 and D39. The D12 of A/A2-1 is replaced by an asparagine in AVD and AVR2, which has been shown to hydrogen bond to the ureido ring of biotin [42] and mutation of the corresponding residue in streptavidin to alanine lowered drastically the biotin affinity of the mutant by disabling hydrogen bonding of the mutated residue to biotin [43]. Based purely on the X-ray structure presented here, the D12 of A/A2-1 seems to be hydrogen bonded (2.8 Å) to the carbonyl oxygen atom of the ureido ring of biotin (Figure 2A), in a similar manner as seen e.g. in the crystal structures of the parental proteins AVD [PDB: 2AVI] and AVR2 [PDB: 1WBI], but where an asparagine, unaffected by pH fluctuation in this regard, is involved in the hydrogen bond. In bulk water, protonation of the side chain of aspartate occurs only at a lower pH (pK_a_ = 3.9). At pH 7, D12 of A/A2-1 may not be able to act as an effective hydrogen bond donor, because of the low pK_a_ value (3.9) of its side chain. A/A2-1 was crystallized at acidic conditions (pH near 4), and hence the X-ray structure of A/A2-1 represents a snapshot of conditions near the pK_a_ of D12, where about 50% of the D12 residues in the crystal would be expected, if exposed to solvent, to be protonated. In the A/A2-1 structure the OD2 oxygen atom of D12 is surrounded by aromatic and nonpolar residues that would act to reduce the effective dielectric constant (in comparison to bulk water), effectively raising the pK_a_ value and increasing the probability of a protonated D12. This would support the formation of a stable hydrogen bond at pH 4, thus explaining the hydrogen bond observed in the crystal structure.

D39 of A/A2-1 forms a salt bridge with R112 in a way similar to that seen in the AVR2 structure [PDB: 1WBI], whereas in AVD the salt bridge is missing (Figure 2B–D). The main-chain nitrogen atom of D39 in A/A2-1 and AVR2, as well as the nitrogen atom of the equivalent residue A39 in AVD, is hydrogen bonded to biotin; the salt bridge is hence indirectly linked to biotin via D39. Moreover, L97 and S99 are in the close vicinity of the salt bridge in A/A2-1 and are equivalent to Q97 and L99 in AVR2 (Figure 1, Figure 2B,D). These differences could explain the slightly different geometry of the salt bridge of A/A2-1 and AVR2. Furthermore, residues T75 and L97 of A/A2-1 and S75 and Q97 of AVR2 are in contact with biotin and may thus directly influence the biotin-binding properties of these proteins, too. T75, L97 and S99 of A/A2-1 originated from AVD (T77, L99 and S101).

The L3,4-loop conformation of the A/A2-1 is highly similar to AVR2 and AVR4 and might at least partially explain the observed high thermal stability. The L3,4-loop is rather flexible in AVD without biotin and upon binding of biotin serves as a lid, locking the biotin-binding site [42]. In the L3,4-loop of AVR2, AVR4 and A/A2-1 (Figure 1), there is a proline residue, which introduces rigidity, and the loop is further stabilized by the intermonomeric salt bridge between D39 and R112 as described above for A/A2-1 and reported earlier for AVR2 and AVR4 [20], [44]. As shown for AVR4 [44], the conformation of the proline-containing L3,4-loop does not change drastically upon biotin binding and the bound biotin is partially exposed to the solvent [44]. The sequence stretch of residues 38–58 represents the β-strand 4 and most of the adjacent L3,4- and L4,5-loops and is likely to be the main source for the increased stability. The sequence region 38–58 is from AVR2 and also found in AVR4, another highly thermostable avidin-related protein found in chicken [45]. Moreover, residues 38–58 have successfully been transferred to AVD, to create an ultra-stable chimeric protein termed ChiAVD with T_m_ = 111.1°C in the absence of ligand [46].

### Selection of a chimeric mutant with improved biotin binding

The chimeric mutants, previously selected by phage display biopanning of the AVD/AVR2 DNA shuffling library against a biotin-coated surface, showed increased biotin dissociation rates, reflecting less-tight biotin binding in comparison to either AVD or AVR2 [15]. These results demonstrated that choosing the optimal parameters during the phage display biopanning process is crucial for the successful selection of high-affinity binders. In order to select for mutants with tighter biotin binding, we modified the biopanning method 1) by adding free biotin to the wash buffer prior to elution, which increased the harshness of the washing conditions, 2) by adding a disulphide bond in the linker between biotin and the coating protein, which allowed cleavage of the linker and therefore ligand-binding independent elution, and 3) by developing a new powerful microplate assay for functional screening of the clones. The phage titers obtained in each biopanning round and the enrichment of phages are shown in Table S1 and Figure S1, respectively. The additional wash step with free biotin removed mutants with high dissociation rate towards biotin and the use of a biotinylation reagent with a cleavable linker ensured the release of even the tightest and acid-resistant binders.

The initial analysis of the expressed protein was made using an avidin-biotin displacement assay (ABD-assay). The assay compares two biotinylated wells that were incubated with cell lysate containing the expressed protein. While one well is incubated with buffer, the other well is incubated with buffer containing biotin. Bound biotin binding proteins are subsequently detected by biotinylated alkaline phosphatase. The ratio between the resulting signals of the two wells gives an indication of the biotin dissociation rate. A total of 22 colonies were selected after the fourth biopanning round for further analysis. AVD and AVR2 were included as controls, however, they gave a response that was not substantially higher than the background. This is due to low expression levels of the parental proteins when expressed from phagemid plasmid and has been observed already earlier [15]. Out of the analyzed mutants, 12 displayed a positive binding response (i.e. an absorbance at 405 nm higher than the background absorbance) and were further analyzed by sequencing. Eleven of these mutants were identical with the previously selected and characterized mutant A/A2-3 [15]. The remaining mutant, named A/A2-B, showed a good binding response and had a high response ratio for the biotin-treated versus untreated wells, thus indicating a slow dissociation from the biotin-functionalized surface (Table 2). The observed response ratio was higher (meaning a lower dissociation rate) than for A/A2-3 [15]. Interestingly, the sequence of A/A2-B only differs at six residue positions from the previously characterized mutant A/A2-1 (Figure 3) [15], however, the dissociation rates of A/A2-1 and A/A2-B were clearly different (Table 2).

**Table 2:** Avidin-biotin displacement assay.

mutant	BSA coating	BTN coating	BTN coating+BTN treatment	ratio between BTN treated and untreated samples
	average^b^±SD	average±SD	average±SD	ratio
background	0.07±0.01	0.07±0.01	0.07±0.01	1.0
AVD (purified)^a^	0.07±0.01	0.18±0.03	0.18±0.03	1.0
AVR2 (purified)	0.07±0.01	0.27±0.05	0.07±0.00	0.3
AVD	0.07±0.00	0.08±0.01	0.07±0.01	0.9
AVR2	0.07±0.01	0.07±0.00	0.07±0.01	1.0
A/A2-1	0.08±0.01	0.14±0.04	0.08±0.01	0.6
A/A2-3	0.12±0.03	1.31±0.64	0.26±0.08	0.2
A/A2-B	0.07±0.01	0.24±0.09	0.31±0.15	1.3

Absorbance measured at 405 nm from samples on differently treated microplate wells are shown. Please note that the background was not subtracted from the reported values.

a 16 µg/ml of bacterial expressed, purified recombinant protein.

b average values and standard deviation calculated from eight independent measurements.

The screening of the selected mutants using the ABD-assay confirmed the tight biotin binding and enabled the selection of efficiently expressing mutants. However, selection of mutants showing high ratios between biotin-treated and non-treated wells has to be done with care. Mutants with low binding response cannot give adequate ratio values due to the high deviation between single measurements (Table 2). In addition, mutants with too low binding response should be avoided, because the low binding response is also an indication for a low expression level, as seen for the parental proteins. Therefore, mutants giving high binding responses and high ratio values should be selected as promising candidates for further characterization. Nevertheless, the improved ligand-binding properties of A/A2-B over A/A2-1 demonstrated the importance of choosing the optimal parameters for biopanning experiments.

### Recombinant production of chimeric mutant proteins and characterization of oligomeric state

The mutants A/A2-1 and A/A2-B and the parental proteins AVD and AVR2 were subsequently produced in *E. coli*, purified using 2-iminobiotin affinity chromatography and identified by denaturing MS. A/A2- B showed higher protein expression levels in bottle cultures (7 mg/l cell culture) as compared to A/A2-1 (5 mg/l cell culture), AVD (3 mg/l cell culture) and AVR2 (2 mg/l cell culture).

The integrity of the oligomeric states was analyzed by native MS as well as by size-exclusion chromatography with on-line static and dynamic light scattering detector (SEC-LS/DLS). These analyses showed that both proteins formed stable tetramers in the presence and absence of biotin (Figures S2 and S3). The experimental molecular masses were in agreement with the theoretical molecular masses of the homotetrameric proteins. In addition, the hydrodynamic radius for the tetramer, calculated from the crystal structure of A/A2-1 [PDB: 4BCS] using the HYDROPRO software [40] was consistent with the experimental data (Table S2). Furthermore, the SEC analysis indicated strong interaction with the column for both mutants, seen by a low UV response and elution in multiple peaks that contain protein species of identical molecular weight (Figure S3A,C). This explains the previously observed lower molecular mass estimate for the A/A2-1 in SEC [15].

### Analysis of biotin binding properties of A/A2-1 and A/A2-B

As observed in the crystal structure, D12 of A/A2-1 hydrogen bonds with the ureido oxygen of biotin in low pH conditions. To confirm the presence of this hydrogen bond at low pH and to see whether D12 has an effect on the biotin-binding affinity, we analyzed the biotin binding by isothermal titration calorimetry (ITC) (Figure S4). Because ITC is not suitable for the determination of accurate dissociation equilibrium constants (K_D_) for high-affinity binding reactions, it was not possible to provide estimates for affinities for the avidin-biotin interaction or for the A/A2-1-biotin interaction at pH 3. However, at pH 7, A/A2-1 showed signs of clearly lower affinity as compared to at pH 3 and a K_D_ estimate of 103 nM was obtained. Overall, the ITC analysis showed that avidin and A/A2-1 differ from each other in terms of the pH-dependency of the biotin binding. The binding enthalpies (ΔH) were not affected by the change in pH (−30 kcal/mol for avidin and −25 kcal/mol for A/A2-1).

**Figure 4:**
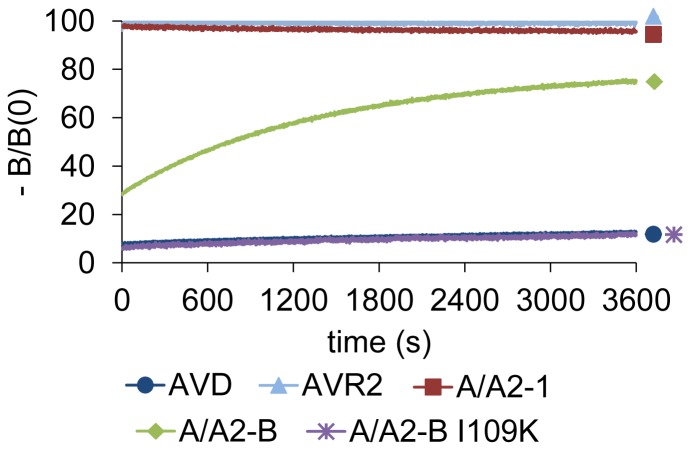
Release of the fluorescence-labeled biotin probe. The biotinylated fluorescence probe BF560 was released from the proteins at 25°C and followed over one hour after addition of free biotin. Please note the immediate release of the fluorescence probe in case of AVR2 and A/A2-1, indicating high dissociation rate.

The observed lowered dissociation rate of mutant A/A2-B as compared to A/A2-1 was confirmed by following the dissociation of the biotin labeled with the fluorescent dye BF560 (Figure 4). At 25°C, AVR2 and A/A2-1 showed very rapid dissociation of BF560-biotin; within seconds after the addition of free biotin, all of the probe was released from the protein, indicating a k_diss_>0.1 s^−1^. In comparison, AVD showed a slow dissociation rate of 0.13·10^−4^ s^−1^; even one hour after the addition of free biotin less than 20% of the probe was released from the protein. Moreover, A/A2-B showed a dissociation rate between that of AVD and AVR2, and clearly lower than A/A2-1. Yet, the dissociation rate (3.08·10^−4^ s^−1^) of A/A2-B measured was about 25-fold faster than that observed for AVD, and 76% of the probe was released from A/A2-B one hour after the addition of free biotin.

It is difficult to predict the factors that are responsible for the lowered dissociation rate of A/A2-B in comparison to AVR2 and A/A2-1. In addition to the already discussed residue 12, two residues that are in contact with biotin may contribute to the binding properties: residue 75, which is threonine in A/A2-1, A/A2-B and AVD but serine in AVR2, and residue 97, which is leucine in A/A2-1, A/A2-B and AVD, but glutamine in AVR2. The presence of a serine residue at position 75 of AVR2 has been reported to increase the size of the biotin-binding pocket and hence decrease the affinity to biotin (Figure 2D) [20]. The additional methyl group on T75, as seen in the A/A2-1 structure, forms multiple hydrophobic interactions with biotin (C6 and S1 atoms) and with F77 (CD1 and CE1 atoms) that would increase the biotin affinity in comparison to S75 in AVR2. In the A/A2-1 structure, the terminal methyl groups of L97 pack against the centrally located C2 and C8 carbon atoms on one face of biotin. Replacement by glutamine in AVR2 negates these nonpolar interactions but the amide side chain is then ideally located to hydrogen bond elsewhere: to the side chains of S75 and R112 (affecting slightly the coplanarity of the salt bridge between D39 and R112) and to the O12 atom of biotin.

Another residue that could affect the dissociation rate of both A/A2-1 and A/A2-B is I109 (see Figure 3). Residue 109 in AVR2 is also isoleucine, however, in AVD and AVR4 the equivalent residue is lysine. When K109I mutation was introduced into AVD or AVR4, it reduced the affinity towards biotin [20] and destabilized those proteins. Conversely, changing isoleucine to lysine at this position in AVR2 increased the thermal stability [20]. To study if this mutation would have a similar effect in A/A2-B, we mutated I109 to lysine and indeed, the mutation further decreased the dissociation rate of A/A2-B complexed with Bf560-biotin to a similar level as seen for AVD (Figure 4).

### High thermal stability of the chimeric mutants

High stability of proteins over a broad pH range is required for many applications. Therefore, the thermal stability of the chimeric avidin mutants was analyzed at varying pH values. In differential scanning calorimetry (DSC) analysis without biotin, A/A2-1 and A/A2-B showed increased thermal stability at all pH values studied (pH 3, pH 7, pH 11) in comparison to AVD; the most clear difference was found at pH 3 (Table 3). Mutation I109K had the stabilizing effect expected and A/A2-B I109K showed the highest thermal stability of all the evaluated proteins, with transition midpoints (T_m_) at least 3°C higher as observed for A/A2-B in all pH values. In the presence of D-biotin, the thermal stability of A/A2-B and A/A2-B I109K exceeded that of AVR2, which may reflect the higher biotin-binding affinity of these two mutants, which was also supported by the ligand dissociation analysis (Figure 4). The pH-dependence of thermal stability, with or without biotin, was apparent for AVD and A/A2-1, whereas A/A2-B and A/A2-B I109K seemed to be less sensitive to changes in the pH. For example, the T_m_ of AVD at pH 7 dropped from 79.9°C to 51.2°C at pH 3 and to 67.7°C at pH 11, whereas the difference between the highest (at pH 7) and lowest (at pH 11) T_m_ value was only 7.2°C for both A/A2-B and A/A2-B I109K (Table 3). The addition of biotin stabilized AVD, A/A2-B and A/A2-B I109K notably, but the thermal stability of A/A2-1 was not substantially affected by biotin binding, as has been previously reported [15]. However, at pH 3, the increase of thermal stability upon D-biotin binding (ΔT_m_) for A/A2-1 was 23.1°C, which is substantially higher than at neutral pH (8.5°C) and in the range observed for A/A2-B (ΔT_m_ = 26.8°C; Table 3). This finding supports the model, where D12 is protonated at low pH conditions and enhances bonding to biotin. Unfortunately, it was not possible to analyze AVR2 at pH 3, because the protein precipitated upon dialysis at this pH, most probably because of the low isoelectric point (pI = 4.7) of AVR2. In general, the thermal stability of A/A2-B and A/A2-B I109K did not drastically change at any of the analyzed conditions.

**Table 3:** Thermal transition midpoints (T_m_) and the stability differences between biotin-bound and biotin-free forms (ΔT_m_) at different pH-values as determined by DSC.

	T_m_ (°C)		ΔT_m_ (°C)
	−BTN	+BTN	
AVD			
pH 3	51.2±1.1	97.5±0.9	46.4
pH 7	79.6±0.4	121.0±0.1	41.3
pH 11	67.7±0.2	109.1±0.1	41.4
AVR2			
pH 3	N.A.^a^	N.A.^a^	
pH 7	95.6±0.1	115.3±0.2	19.6
pH 11	76.8±2.1	99.0±2.1	22.2
A/A2-1			
pH 3	80.4±0.6	103.5±0.6	23.1
pH 7	90.5±0.3	99.9±0.2	8.5
pH 11	72.2±3.2	83.8±0.7	11.6
A/A2-B			
pH 3	89.3±0.3	116.4±0.1	27.1
pH 7	92.5±0.1	119.3±0.5	26.8
pH 11	85.3±0.9	109.7±1.9	24.4
A/A2-B I109K			
pH 3	92.0±0.1	123.5±0.0	31.5
pH 7	96.3±0.1	126.7±0.1	30.4
pH 11	89.1±0.8	113.8±0.5	24.7

Average values and standard deviation were calculated from two independent measurements. Values for A/A2-B I109K were derived from three independent measurements.

a AVR2 precipitated completely upon dialysis against pH 3 buffer.

**Figure 5:**
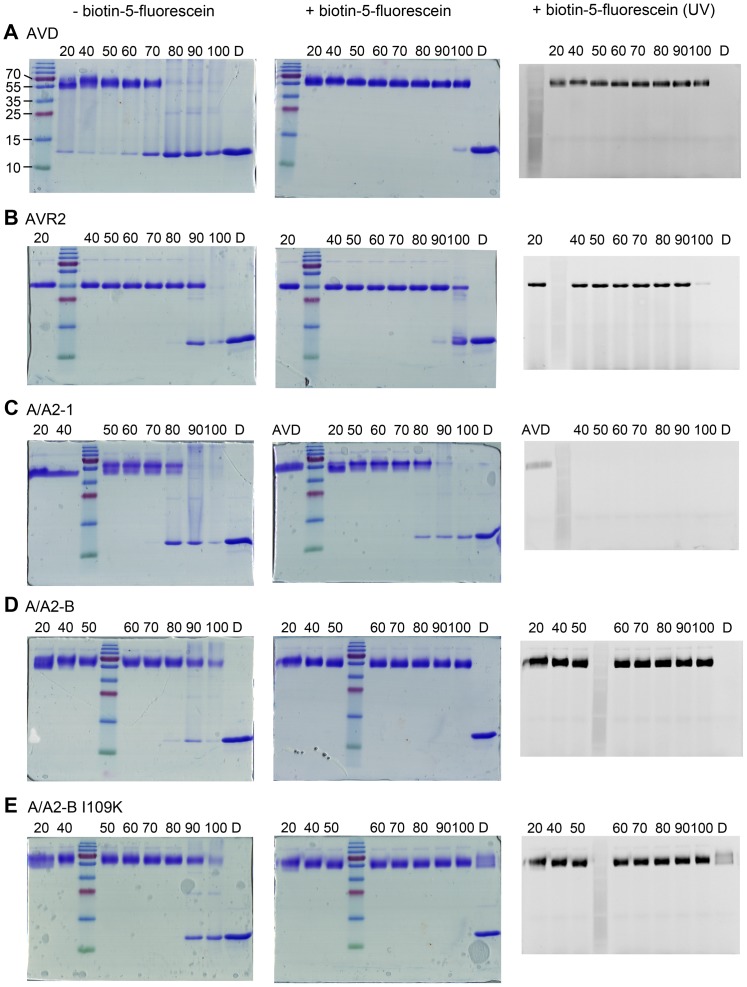
Tetramer stability of analyzed proteins as determined by SDS-PAGE stability assay. The proteins were incubated for 20(left panel) or presence (middle and right panel) of biotin-5-fluorescein. A. AVD, B. AVR2, C. A/A2-1, D. A/A2-B, E. A/A2-B I109K. The gels were stained with Coomassie Brilliant Blue staining. The gels in the presence of biotin-5-fluorescein were imaged under UV light before staining (right panel). Numbers indicate the temperature in °C at which the samples were incubated. D: protein sample incubated at 100°C for 20 min in the presence of 2% SDS and 0.5% β-mercaptoethanol. AVD in C refers to AVD in the presence of biotin-5-fluorescein incubated at 20°C.

The thermal stability of the proteins was further analyzed by a SDS PAGE stability assay (Figure 5). The stability of the tetramer was followed by incubation of the proteins in the absence and presence of biotin-5-fluorescein at different temperature and subsequent analysis on a 15% acrylamide-bisacrylamide gel [35], [36]. The use of biotin-5-fluorescein allows the detection of active biotin binding proteins when exposed to UV light [36]. In the absence of biotin, AVD was the least stable of the analyzed proteins and was completely disassembled into monomers at 80°C, which was in line with the results from the DSC analysis (Figure 5A). Both chimeric mutants showed higher thermal stabilities in comparison to AVD, with A/A2-B being substantially more stable than A/A2-1. Both, A/A2-B and A/A2-B I109K were found to have tetramers present even after incubation at 100°C (Figure 5D,E). In the presence of biotin, A/A2-B I109K was even present as a functional tetramer protein in denaturing conditions, i.e. when boiled in the presence of SDS and β-mercaptoethanol (Figure 5E). The SDS PAGE stability assay supported the low biotin-binding affinity observed for A/A2-1. No bound biotin-5-fluorescein was visible under UV light for A/A2-1, indicating that the biotin had dissociated completely during the run of the SDS PAGE (approximately 1 h, Figure 5D).

In conclusion, the crystal structure of A/A2-1 revealed structural details that disclose the details behind the previous physicochemical observations. Improved phage display selection of the DNA-shuffled AVD/AVR2 library resulted in a chimeric mutant (A/A2-B) with higher thermal stability than that observed for the parental proteins and a lower biotin dissociation rate than that measured for the previously reported mutant, A/A2-1. A/A2-B displayed high stability at a broad pH range and the additional mutation I109K improved both biotin-binding affinity and thermal stability. The highly stable chimeric avidins reported here may help in the design of improved, higher stability engineered avidins such as highly-stable steroid-binding avidins [47].

## Supporting Information

Figure S1 **Phage enrichment over four rounds of biopanning.** Output/input ratio was determined from the amount of phages added in each biopanning round (input) and the phages obtained after each biopanning round (output), which can be found from Table S1.
(TIF)

Figure S2 **Native-MS analysis of analyzed proteins.** Native mass spectra of 10 µM protein (monomer concentration) in 10 mM ammonium acetate, pH 6.9. A. A/A2-1, B. A/A2-1+BTN, C. A/A2-B, D. A/A2-B+BTN, E. AVD and F. AVD+BTN. Te corresponds to the protein tetramer and numbers denote different charge states.
(TIF)

Figure S3 **SEC-LC analysis.** Proteins were run in phosphate buffer containing 650 mM sodium chloride on a Superdex75 column at 12°C. UV-VIS absorbance at 280 nm (UV), static light scattering (LS) and dynamic light scattering (DLS) of the eluting protein were recorded. The left Y-axis shows the scale of the UV and LS signal intensities. Molecular weight (MW) and hydrodynamic radius were calculated from the LS and DLS signal, respectively, using BSA for the calibration of the LS detector. The right Y-axis shows the scale for MW and hydrodynamic radius. A. A/A2-1, B. A/A2-1 in the presence of 3-fold molar excess of biotin, C. A/A2-B, D. A/A2-B in the presence of 3-fold molar excess of biotin.
(TIF)

Figure S4 **ITC analysis of A/A2-1 and AVD at different pH values.** The thermograms of biotin titration to protein were recorded at pH 3 and pH 7 at 40°C. The top panels display the raw ITC data, while the bottom panels display the binding isotherm derived from integrated heats. The affinities towards biotin were too high to accurately calculate the dissociation equilibrium constant from the integrated heats. A. Biotin titration to AVD at pH 3, B. biotin titration to AVD at pH 7, C. biotin titration to A/A2-1 at pH 3 and D. biotin titration to A/A2-1 at pH 7.
(TIF)

Table S1 **Phage titers during biopanning.** The enrichment was calculated from the ratio between output and input titer.
(DOC)

Table S2 **Molecular weight and hydrodynamic radius obtained by SEC-LS and DLS analysis.**
(DOC)
