# Supplementary figures and images for: A Novel Chimeric Avidin with Increased Thermal Stability Using DNA Shuffling
Source: PLoS One. 2014 Mar 14;9(3):e92058. doi: 10.1371/journal.pone.0092058 (PMC3954883; doi:10.1371/journal.pone.0092058)

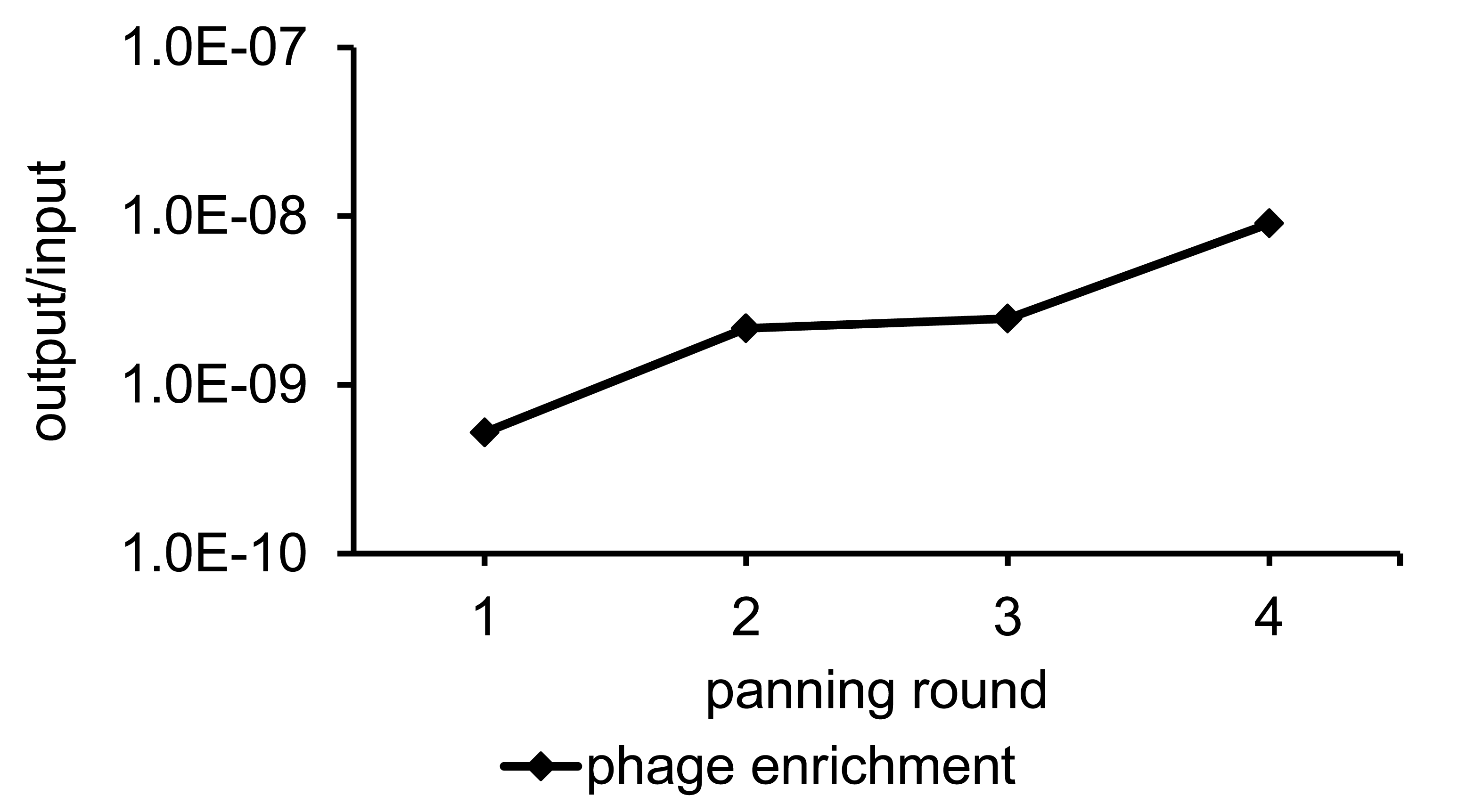

Supplement: Figure S1 — Phage enrichment over four rounds of biopanning. Output/input ratio was determined from the amount of phages added in each biopanning round (input) and the phages obtained after each biopanning round (output), which can be found from Table S1. (TIF) [file pone.0092058.s001.tif]

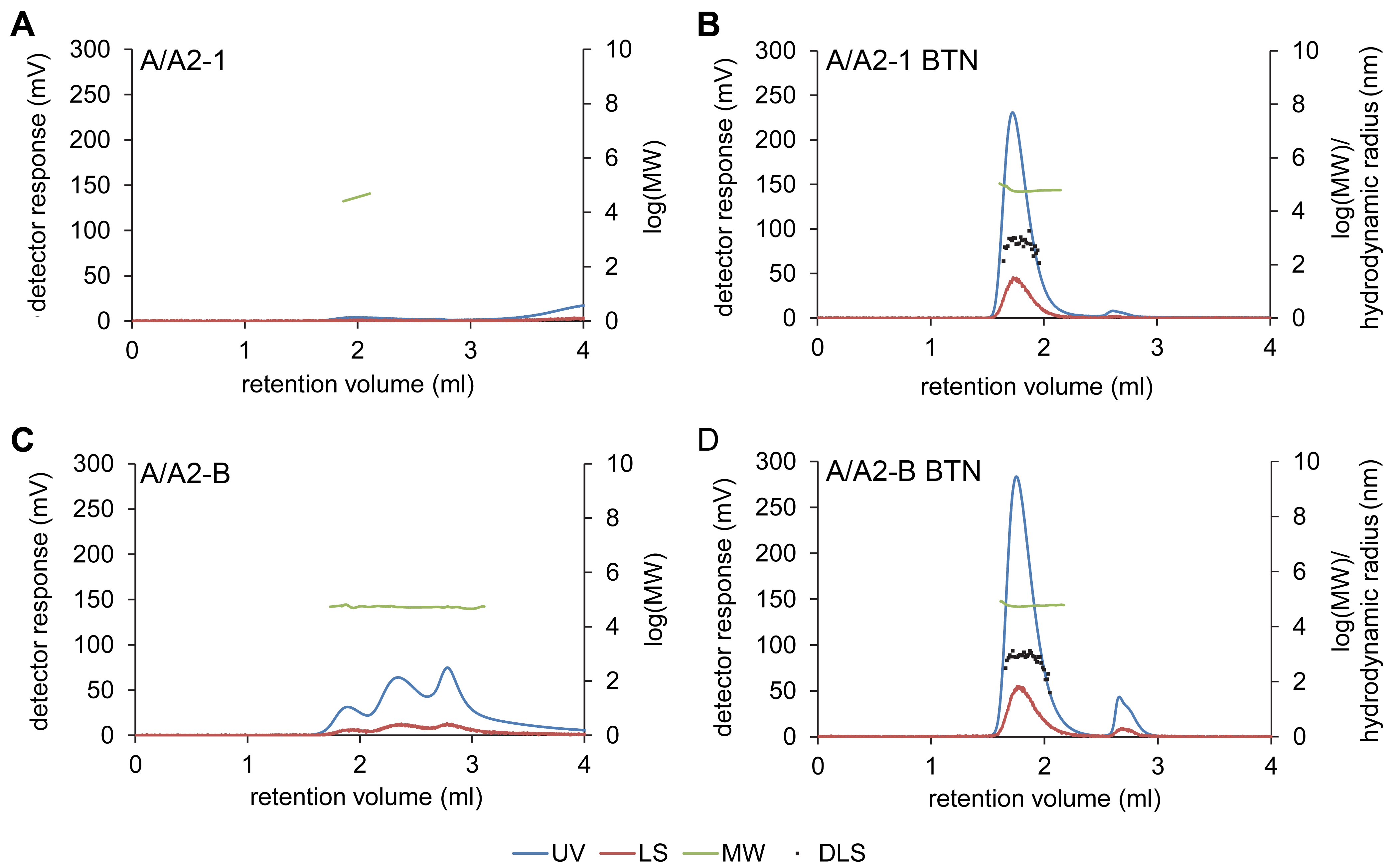

Supplement: Figure S3 — SEC-LC analysis. Proteins were run in phosphate buffer containing 650 mM sodium chloride on a Superdex75 column at 12°C. UV-VIS absorbance at 280 nm (UV), static light scattering (LS) and dynamic light scattering (DLS) of the eluting protein were recorded. The left Y-axis shows the scale of the UV and LS signal intensities. Molecular weight (MW) and hydrodynamic radius were calculated from the LS and DLS signal, respectively, using BSA for the calibration of the LS detector. The right Y-axis shows the scale for MW and hydrodynamic radius. A. A/A2-1, B. A/A2-1 in the presence of 3-fold molar excess of biotin, C. A/A2-B, D. A/A2-B in the presence of 3-fold molar excess of biotin. (TIF) [file pone.0092058.s003.tif]

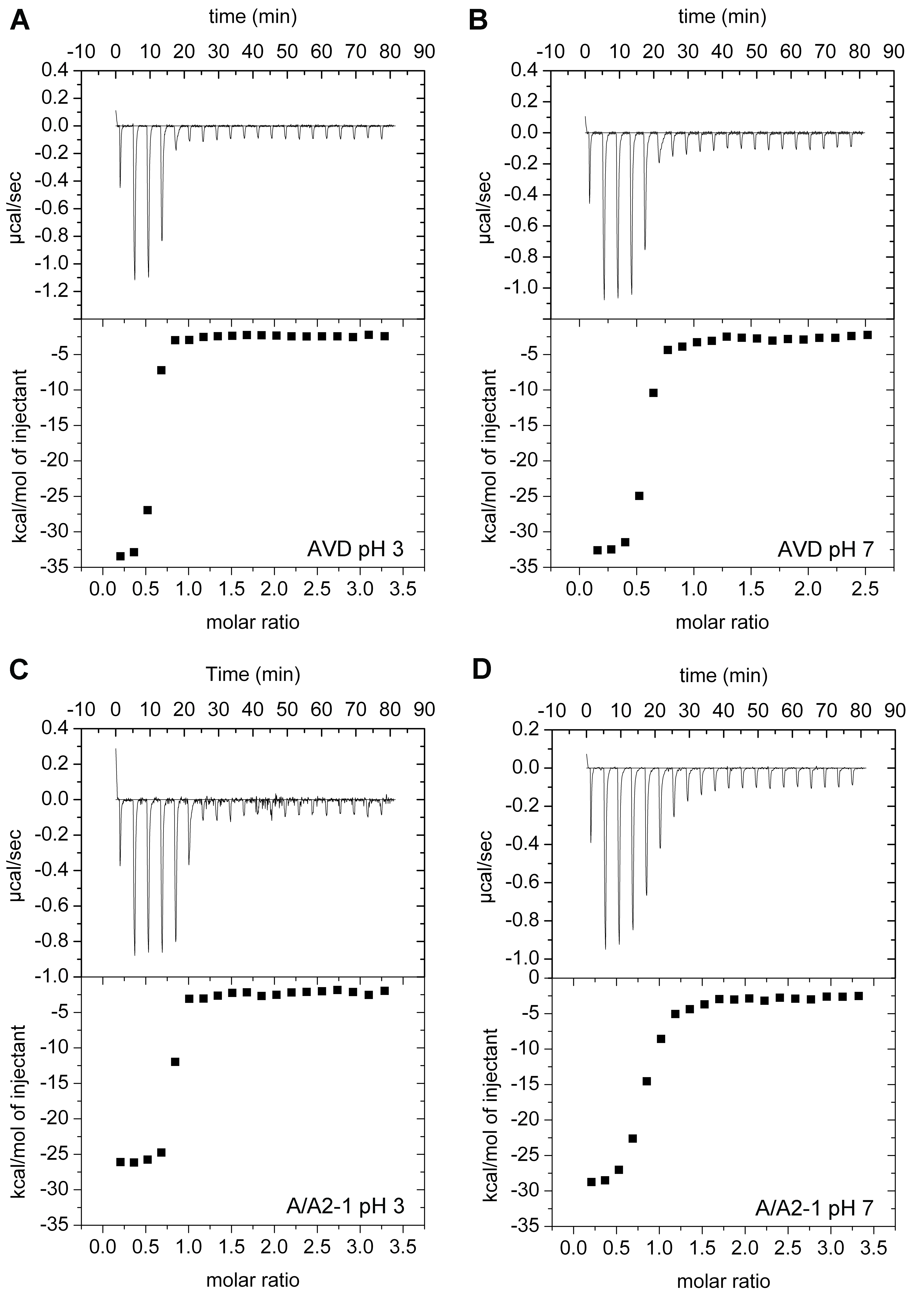

Supplement: Figure S4 — ITC analysis of A/A2-1 and AVD at different pH values. The thermograms of biotin titration to protein were recorded at pH 3 and pH 7 at 40°C. The top panels display the raw ITC data, while the bottom panels display the binding isotherm derived from integrated heats. The affinities towards biotin were too high to accurately calculate the dissociation equilibrium constant from the integrated heats. A. Biotin titration to AVD at pH 3, B. biotin titration to AVD at pH 7, C. biotin titration to A/A2-1 at pH 3 and D. biotin titration to A/A2-1 at pH 7. (TIF) [file pone.0092058.s004.tif]
