# Supplementary material for: A Novel Chimeric Avidin with Increased Thermal Stability Using DNA Shuffling
Source: PLoS One. 2014 Mar 14;9(3):e92058. doi: 10.1371/journal.pone.0092058 (PMC3954883; doi:10.1371/journal.pone.0092058)
Supplement: Table S1 — Phage titers during biopanning. The enrichment was calculated from the ratio between output and input titer. (DOC) [file pone.0092058.s005.doc]

**Supporting Table S1:** Phage titers during biopanning. The enrichment was calculated from the ratio between output and input titer.

| **panning round** | **input titer** | **output titer** | **output/input** |
| --- | --- | --- | --- |
|  | (x10^14^ cfu/ml) | (x10^5^ cfu/ml) | (x10^-9^ cfu/ml) |
| 1 | 46 | 24 | 0.5 |
| 2 | 3.7 | 8.0 | 2.2 |
| 3 | 0.3 | 0.8 | 2.5 |
| 4 | 0.2 | 1.9 | 9.0 |
