# Supplementary material for: A Novel Chimeric Avidin with Increased Thermal Stability Using DNA Shuffling
Source: PLoS One. 2014 Mar 14;9(3):e92058. doi: 10.1371/journal.pone.0092058 (PMC3954883; doi:10.1371/journal.pone.0092058)
Supplement: Table S2 — Molecular weight and hydrodynamic radius obtained by SEC-LS and DLS analysis. (DOC) [file pone.0092058.s006.doc]

**Supporting Table S2.** Molecular weight and hydrodynamic radius obtained by SEC-LS and DLS analysis

| **protein sample** | **elution volume** | **molecular weight** | | **hydrodynamic radius^a^** | |
| --- | --- | --- | --- | --- | --- |
|  | **(ml)** | **(kDa)** | | **(nm)** | **(nm)** |
|  | **SEC-LS** | **SEC-LS** | **theoretical** | **SEC-DLS** | **DLS^b^** |
| BSA | 1.57 | 66.5 | 66.4 | 3.74 |  |
| AVR2 | 1.70 | 54.6 | 55.9 | 3.07 | 3.57 |
| AVR2 BTN | 1.69 | 56.8 | 56.9 | 3.11 | 3.50 |
| AVD | 1.62 | 50.2 | 57.4 | 3.14 | 3.52 |
| AVD BTN | 1.70 | 63.2 | 58.4 | 3.09 | 3.53 |
|  | 2.70 | 73.1 | 58.4 | N.A. |  |
| A/A2-1 | 1.96 | 51.9 | 55.6 | N.A. | 3.39 |
| A/A2-1 BTN | 1.72 | 57.3 | 56.6 | 2.83 | 3.26 |
|  | 2.61 | 57.2 | 56.6 | N.A. |  |
| A/A2-B | 1.89 | 55.3 | 55.5 | N.A. | 3.38 |
|  | 2.34 | 52.3 | 55.5 | N.A. |  |
|  | 2.78 | 50.9 | 55.5 | N.A. |  |
| A/A2-B BTN | 1.75 | 56.7 | 56.4 | 2.90 | 3.25 |
|  | 2.66 | 57.9 | 56.4 | N.A. |  |

N.A.: DLS signal too low to obtain values

^a^The hydrodynamic radius for the tetramer of A/A2-1 was 3.145 nm, as calculated based on the PDB coordinates [4BCS]

^b^determined with batch DLS at protein concentration of 1 mg/ml
